# Pregnancy- and Lactation-Associated Osteoporotic Vertebral Fracture: A Case Report

**DOI:** 10.7759/cureus.31322

**Published:** 2022-11-10

**Authors:** Aarti Jadhav, Shalik Jadhav, Anuj Varma

**Affiliations:** 1 Medicine, Jawaharlal Nehru Medical College, Datta Meghe Institute of Medical Sciences, Wardha, IND; 2 Medicine, Yashoda Hospital, Secunderabad, Hyderabad, IND

**Keywords:** kyphosis, pregnancy, fish mouth vertebra, compression, fracture, osteoporosis

## Abstract

The report details an instance of a 29-year-old postpartum female who presented to us after six months of delivery and had symptoms of severe back pain since the sixth month of pregnancy. The pain was located in the lower thoracic and lumbar regions. It got aggravated by standing or walking and got relieved by lying down. The pain was radiating from the back to both lower limbs. On examination, forward rounding of the upper back, i.e., kyphosis, was seen, for which the patient was advised to get an X-ray, which was suggestive of severe osteopenia with wedging of L1-L4 vertebrae. For a thorough assessment, MRI was performed, which confirmed exaggerated kyphosis at L1 and L2 with mild scoliosis in the thoracolumbar region. The patient was advised to take calcium and vitamin D supplements with bisphosphonates. A monthly checkup was advised. After four months, the symptoms of the patient were partially eased. On investigation, the serum calcium and phosphorous levels were found to be within the normal range.

## Introduction

Osteoporosis is an asymptomatic disorder in which the density of normal mineralized bone is decreased [1]. The loss of bone mass leads to the loss of strength, so a trivial trauma can cause a fracture. Older age, female gender, menopause, low levels of calcium and vitamin D, and a sedentary lifestyle are some of the major risk factors for osteoporosis. The intake of nutrition along with calcium and vitamin D supplements plays an important role in achieving adequate bone density. Clinically, osteoporosis in postmenopausal females is the most common form of bone loss, but it is relatively uncommon in young pregnant females. In pregnancy, calcium from the mother is transferred to the fetus for its skeletal development, which leads to a decreased calcium level in pregnant females. Early diagnosis and treatment of this condition are necessary to prevent further fractures or complications. The evaluation of serum calcium, serum phosphate, and bone mineral density (BMD) by densitometry is suggestive of multiple fractures of lumbar vertebrae, which indicate osteoporosis [2].

## Case presentation

A 29-year-old postpartum female came with complaints of severe back pain since six months of pregnancy, which was exacerbated two months after delivery. The pain was located in the lower thoracic and lumbar regions and radiated to both lower limbs. It gets aggravated by standing or sitting and gets relieved by lying down. She even needs support for walking. Her obstetric score was G2P2L1 with a history of miscarriage at four months of her pregnancy two years ago, after which she conceived again and had a healthy male child who she was feeding. On physical examination, her blood pressure was 110/70 mmHg, and her pulse was 72/minute. Her height was 139.12 cm, her weight was 45 kg, and her body mass index was 20.72 kg/m^2^. Her thyroid gland was normal. No pallor, icterus, and feet edema were noted.

On examination, kyphosis was present with tenderness over the lower back region. There were no history of any sensory impairment; no history of falls or trauma to the spine; no past history of any bone fracture, endocrine disease, and connective tissue disorder; and no evidence suggestive of malabsorption. Laboratory investigations were carried out, the details of which are shown in Table 1.

**Table 1:** Investigation test and result with normal range MCV: mean corpuscular volume; ESR: erythrocyte sedimentation rate

Test	Result
Hemoglobin (Hb)	10.4 g/dl (normal range­ for postpartum female: 7-15 g/dl)
MCV	74
Total leukocyte count	5000/mm^3^
Serum alkaline phosphatase	382.2 U/L (normal range for female: 240 U/L)
ESR	05 mm (normal range for female: 0-22 mm)
Serum 25-hydroxy vitamin D	28.80 ng/mL (normal range: 30-100 ng/mL)
Serum parathyroid hormone (PTH)	21.4 pg/mL (normal range for adult: 15-65 pg/mL)
Serum calcium	7.8 mg/dl (normal range for adult: 8.6-10.3 mg/dl)
Serum phosphorus	5.4 mg/dl (normal range: 2.7-4.5 mg/dl)
Hb electrophoresis	"AA" pattern
Thyroid-stimulating hormone (TSH)	2.73 µIU/mL (normal range: 0.35-5.58 µIU/mL)
Luteinizing hormone	2.8 µIU/mL (normal range: 0.1-6.0 µIU/mL)
Estrogen	51.23 pg/mL (normal range: 150-350 pg/mL)
Vitamin B12	800 pg/mL (normal range: 160-950 pg/mL)
Serum iron	80 mcg/dl (normal range: 60-140 mcg/dl)
C-reactive protein (CRP)	8 mg/dl (normal range: less than 10 mg/dl)
Serum creatinine	0.8 mg/dl (normal range: 0.5-1.0 mg/dl)
Urine test	No abnormal pathological finding
24-hour urine calcium loss	2.6 mg/kg/day (normal range: 2-3 mg/kg/day)
Total serum cholesterol	3 mmol/L (normal range: less than 5 mmol/L)
Parathyroid hormone (PTH)	60 pg/mL (normal range: 15-65 pg/mL)
Serum protein electrophoresis (SPEP)	7.4 g/dl (normal range: 6.4-8.3 g/dl)
Serum immunoelectrophoresis (SIEP) IgA	230 mg/dl (normal range: 82-470 mg/dl)
Serum immunoelectrophoresis (SIEP) IgG	1220 mg/dl (normal range: 694-1760 mg/dl)
Serum immunoelectrophoresis (SIEP) IgM	198 mg/dl (normal range: 50-398 mg/dl)

**Figure 1:**
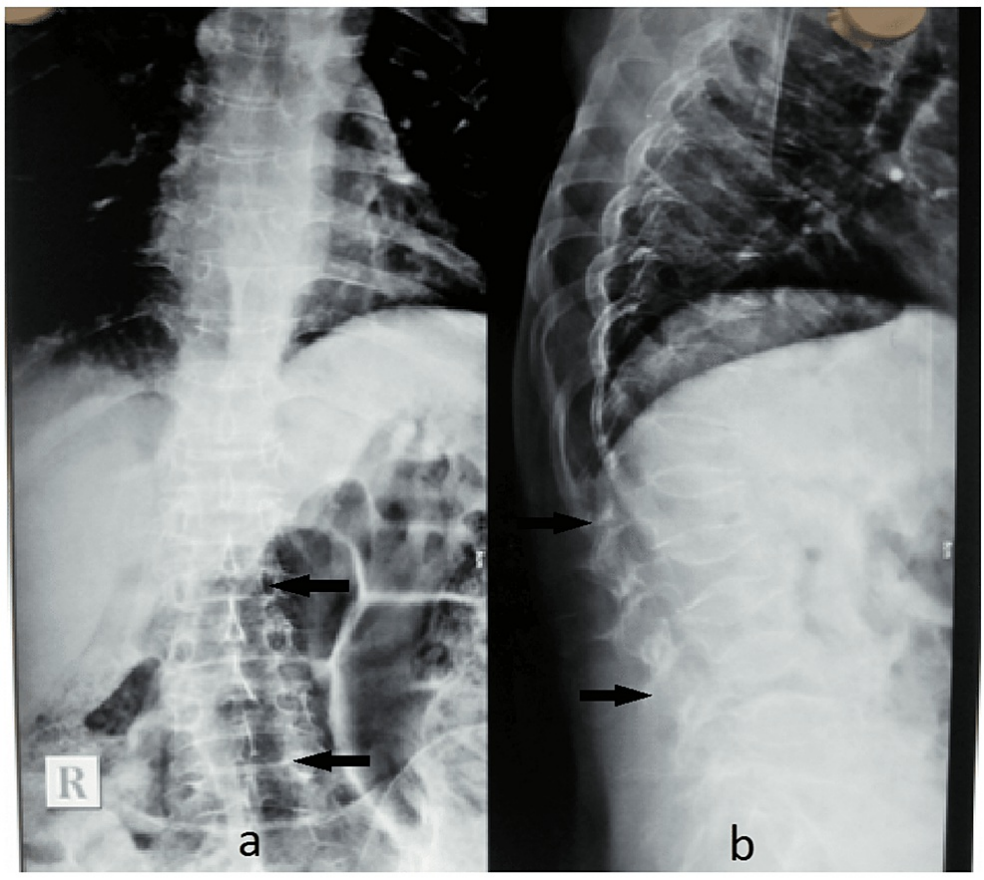
Plain X-ray of the lumbosacral spine a) Anteroposterior view; b) lateral view. The arrows indicate severe osteopenia with wedging of L1-L4 vertebrae

The patient was advised to have a plain X-ray of the lumbosacral spine, which revealed severe osteopenia with wedging of the L1-L4 vertebrae (Figure 1).

**Figure 2:**
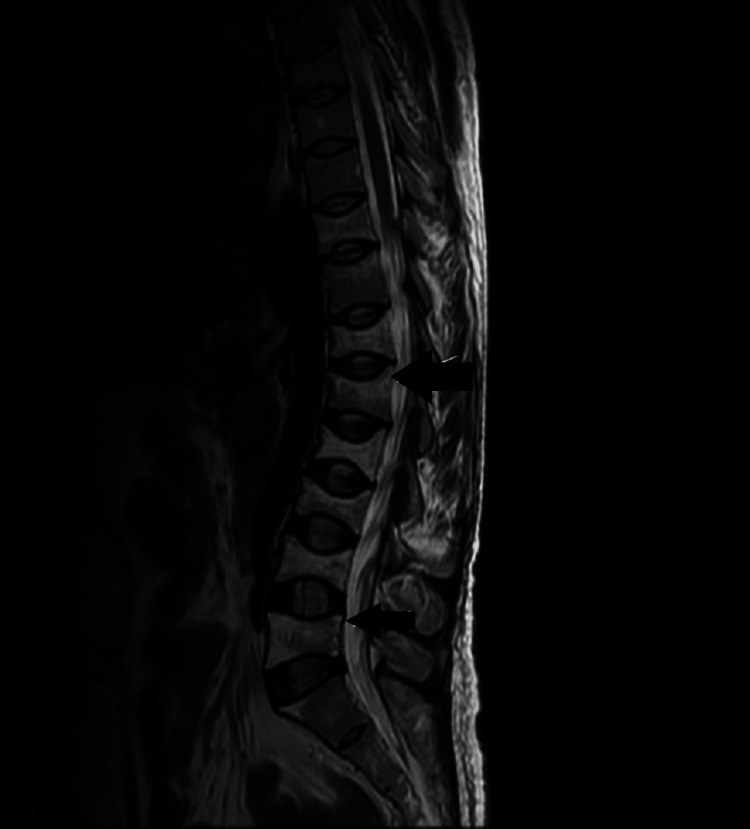
MRI of the spine in sagittal view The arrows indicate the biconcave appearance of T10-T12 vertebrae and all five lumbar vertebrae, fatty marrow replacement in all the lumbar vertebrae and T10-T12 vertebrae, exaggerated kyphosis at L1 and L2, mild scoliosis at the thoracolumbar spine, and increased convexity to the right

MRI of the lumbosacral spine reveals biconcave appearance and fatty marrow replacement of T10-T12 vertebrae and all five lumbar vertebrae, exaggerated kyphosis at L1 and L2 levels, mild scoliosis at the thoracolumbar spine, and increased convexity to the right (Figure 2).

Tables 2-3 show the results of a digital dual-energy X-ray absorptiometry (DEXA) scan.

**Table 2:** Assessment of the axial skeleton Digital dual-energy X-ray absorptiometry (DEXA) scan finding of the axial skeleton with anteroposterior (AP) spine, spine region, bone mineral density, adult T-score, matched to age Z-score, and fracture risk

Site	Region of the spine	Bone mineral density	Adult T-score	Matched Z-score of age	Risk of fracture
AP spine	L1	0.441	-5.2	-5.2	High
	L2	0.466	-5.4	-5.4	High
	L3	0.453	-5.6	-5.6	High
	L4	0.303	-6.8	-6.8	High

**Table 3:** Appendicular skeleton Digital dual-energy X-ray absorptiometry (DEXA) scan findings of the appendicular skeleton with the site, spine region, bone mineral density, adult T-score, matched Z-score of age, and risk of fracture

Site	Region of the spine	Bone mineral density	Adult T-score	Matched Z-score of age	Risk of fracture
Femur	Left neck	0.445	-3.8	-4.3	High
	Right neck	0.388	-4.3	-4.8	High

Based on radiological and magnetic resonance findings, multiple fractures of the vertebrae were found, which is suggestive of pregnancy- and lactation-induced osteoporotic vertebral fracture. There was no evidence of other secondary causes such as endocrine, liver, kidney, genetic, or autoimmune disease.

Following the diagnosis, the patient was advised 2.5 mg of bromocriptine for the cessation of breastfeeding with 600 mg of calcium twice a week (BID) and vitamin D (cholecalciferol 6000 international units) once a week for 10 months, as well as bisphosphonate (alendronic acid) 70 mg pill weekly. After a two-month follow-up, the patient improved symptomatically, and her back pain was reduced. After four months, her serum calcium was 9.4 mg/dl, and her phosphorus was 3.5 mg/dl (within the normal range). Combined calcium and vitamin D supplementation resulted in an increase in lumbar BMD.

## Discussion

Osteoporosis is characterized by the loss of bone mass primarily in the pelvis, femoral neck, and axial skeleton. The radiological findings include abnormalities in the porosity of the bone, trabecular pattern, and vertebral body (also called biconcave fish mouth vertebrae) [3]. It might potentially result in a pathological fracture. The metabolic illness of osteoporosis is linked to several other ailments [4].

The incidence of fragility fracture in pregnancy is rare. The exact cause of pregnancy- and lactation-related osteoporosis is yet to be determined. Some contributing factors can be low levels of calcium due to increased demand for fetal growth and development, a low level of estrogen during pregnancy, and an immature ovarian follicle during lactation resulting in the loss of bone mineral density. Increased mammary gland production of a protein linked to parathyroid hormone during breastfeeding may cause bone resorption. Other factors such as preterm labor and pregnancy-induced hypertension also play a significant role [5]. A secondary cause of osteoporosis may exist. As a result, it's crucial to learn about a patient's background, including any operations or pharmacological treatments they may have had in the past, and to perform endocrine tests for conditions involving hyperparathyroidism, hyperthyroidism, and Cushing's syndrome [6].

Although it is unclear if pregnancy affects bone metabolism, the fetus needs calcium for bone development throughout the third trimester of pregnancy. Even during breastfeeding, a daily average of 200-250 mg of calcium is delivered to the fetus. As the number of pregnancies and the length of pregnancy increase, calcium and vitamin D levels decrease. The consumption of calcium in the postpartum amenorrhea period contributes to pregnancy- and lactation-associated osteoporosis [7].

Calcium and vitamin supplements, as well as bromocriptine for breastfeeding cessation, are the mainstays [8]. Additionally, bisphosphates are also recommended as they are more effective, and nowadays, teriparatide, a human recombinant parathormone, is also available for the treatment of osteoporotic fracture [9]. Breastfeeding cessation and supplementation boost lumbar bone mineral density (BMD) by 6% in eight months and 9.5% in 2-4 years. Lumbar BMD increased by 5%-15% after adding bisphosphonates [10].

## Conclusions

Osteoporosis is a bone disorder commonly affecting the elderly and postmenopausal females. Despite its rarity, we conclude that pregnancy- and lactation-associated osteoporosis should be considered as a differential diagnosis in pregnant or postpartum females experiencing chronic back pain. Since it might result in vertebral or peripheral fractures, causing a decrease in bone mineral density with limited mobility and a reduction in height, it also affects females' mental and physical health and decreases their quality of life. Early diagnosis and treatment are essential for pregnant females to avoid the severity of osteoporosis. Investigations such as radiography and densitometry are helpful in the confirmation of diagnosis. The treatment of this disease with calcium, vitamin D, and antiresorptive medications may be effective with complimentary bed rest, a healthy diet, and exercise.
